# Is There a “Non-Motor Effect” of Botulinum Toxin Treatment in Cervical Dystonia in Addition to Its Effects on Motor Symptoms?

**DOI:** 10.3390/toxins17080396

**Published:** 2025-08-06

**Authors:** Małgorzata Dudzic, Anna Pieczyńska, Artur Drużdż, Anna Rajewska, Katarzyna Hojan

**Affiliations:** 1 Department of Neurology, Joseph Strus Municipal Hospital in Poznan, 61-285 Poznan, Poland; adruzdz@szpital-strusia.poznan.pl (A.D.); aniafr1@gmail.com (A.R.); 2 Department of Occupational Therapy, Poznan University of Medical Sciences, 60-781 Poznan, Poland; apieczynska@ump.edu.pl; 3 Department of Rehabilitation, Greater Poland Cancer Centre, 61-866 Poznan, Poland; 4 Neurorehabilitation Ward, Greater Poland Provincial Hospital, 60-479 Poznan, Poland

**Keywords:** cervical dystonia, non-motor symptoms, cognitive impairment, depression, anxiety, social anxiety, sleep disturbances, botulinum toxin type A, optimization of results

## Abstract

The efficacy of botulinum toxin A (BoNT) in alleviating motor symptoms of cervical dystonia (CD) has been well established, and it is the treatment of choice in this disease. Lately, the effect of BoNT on non-motor symptoms (NMS) such as cognitive function, depression, anxiety, pain, and sleep disturbance has been observed in patients with CD. A comprehensive clinical and functional assessment of motor (dystonia severity, gait) and non-motor symptoms (cognitive functions, depression, anxiety, sleep, and pain) has been performed in a total of 34 adult patients with cervical dystonia before and after BoNT treatment. Results have also been compared to a control group. Significant improvements in the scales assessing dystonia severity have been observed, which is in line with previous studies on the effect of BoNT on motor symptoms in dystonia. Interestingly, the results also clearly indicate that BoNT has a positive effect on NMS. Among the studied non-motor domains, depression and cognitive functions improved the most after the treatment procedure. The study highlights the potential of BoNT to positively influence non-motor symptoms in patients with cervical dystonia, although its effect on various NMS is not equal.

## 1. Introduction

Cervical dystonia is a neurological condition that is characterized by involuntary neck muscle contractions, resulting in abnormal head and neck movements and postures [1]. It is well understood that motor symptoms have a substantial impact on an individual’s ability to engage in daily activities. These symptoms are often so troublesome that, in the absence of treatment, they result in disability. In addition to the core motor features, non-motor symptoms are prevalent in patients with CD and have been demonstrated to significantly impact quality of life [2,3,4]. The NMS may present with a range of symptoms, including cognitive impairment, depression, anxiety, sleep disturbances, and pain, among the most common [5].

Understanding the complex interrelationships between individual motor symptoms and non-motor symptoms is challenging and requires a detailed analysis of the pathomechanism of the disease and the accompanying symptoms. Adverse postures, muscle spasms, and head shaking can considerably alter body image, leading to a diminished self-esteem and social isolation. The presence of persistent pain that frequently accompanies cervical dystonia has been demonstrated to trigger depressive symptoms and cause decreased cognitive functioning.

Since cervical dystonia is a network disorder characterized by the dysregulation of sensorimotor axes within the brain, which are also involved in cognitive and mood regulation processes, a theory has also been postulated that non-motor symptoms can result from the disease itself. Thus, individual non-motor symptoms can either result from or influence basic symptoms; as well, motor and non-motor symptoms can exacerbate each other.

The therapeutic efficacy of the botulinum toxin in alleviating the motor symptoms of cervical dystonia has been well established since the 1980s [6], while its impact on NMS remains uncertain [7,8]. Recent studies have indicated that the botulinum toxin, a substance primarily used to treat pathological muscle contractions, may also influence certain NMS [9]. Emerging evidence suggests that BoNT, beyond its well-established neuromuscular functions, may exert central neuromodulatory effects relevant to these symptoms. According to recent reviews [10,11], local injection of BoNT has demonstrated clinically meaningful antidepressant effects in individuals with major depressive disorder, with response rates comparable to those of conventional pharmacological treatments. Following peripheral injection, BoNT has been shown to undergo retrograde axonal transport and influence central neural circuits, as evidenced by changes in cortical excitability, sensorimotor integration, and functional connectivity in human studies [12]. In animal models of Parkinson’s disease, intrastriatal BoNT administration has led to improvements in anxiety- and depression-like behaviors [13], while in cervical dystonia, clinical observations indicate potential benefits on mood and cognitive functions [9,14,15]. Moreover, recent imaging studies suggest that sensorimotor network connectivity may be restored by BoNT treatment in patients with dystonia [16]. These findings support a broader therapeutic role for BoNT, potentially addressing both motor and non-motor manifestations through central mechanisms that require further investigation.

 The aim of this study was to determine if there is a “Non-Motor Effect” of BoNT treatment in cervical dystonia. In order to accomplish this objective, a comprehensive assessment of motor and non-motor symptoms in patients with cervical dystonia treated with BoNT has been performed.

## 2. Results

### 2.1. Demographics and Baseline Characteristics

**Figure 1:**
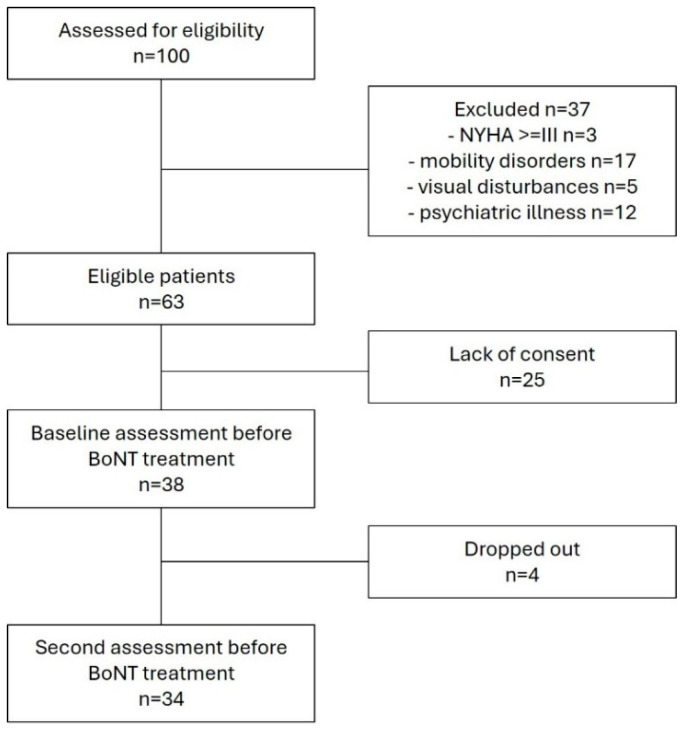
The flow of the participants through the study.

Based on recruitment criteria, 100 patients were assessed for eligibility of the study. Out of the 63 patients who met the study’s inclusion criteria, 25 declined to participate and 4 dropped out during the follow-up assessment (due to missing the follow-up visit, n = 1 or refusal to continue participation in the study, n = 3). The final number of subjects included in the study was 34. The flow of the participants in the study is presented in the flow chart in Figure 1.

After the study group was established, an age- and gender-matched control group was recruited. The demographic characteristics of the study and control group are presented in Table 1.

Most of the patients were treated with onabotulinum toxin or abobotulinum toxin. Three out of thirty-four patients experienced mild side effects (dysphagia, pain in the injection site). The treatment characteristics of the study group are presented in Table 2.

Both study groups were analyzed in terms of cognition, depression, anxiety disorders, sleep disturbances, and gait performance. The study group demonstrated significant differences in the studied parameters compared to the control group, as anticipated. The only parameter in which there was no significant difference (*p* = 0.082) between the groups was sleep disturbances. The results of the clinical assessment of the study group and control group are presented in Table 3.

The results of the baseline assessment of CD patients were compared with the scores in the second assessment to evaluate the effect of BoNT on motor and non-motor symptoms of cervical dystonia (Table 4).

### 2.2. Functional Assessment

The functional assessment consisted of a neurological examination with the assessment of the dystonic pattern according to the Col-Cap concept, TWSTRS (Toronto Western Spasmodic Torticollis Rating Scale) assessment, and mobility and gait performance before and 4–6 weeks after botulinum toxin treatment. The total TWSTRS score as well as severity, disability, and pain subscales improved significantly at the second assessment compared to the baseline assessment (*p* < 0.0001 in total score and each subscale). Parameters reflecting mobility: 10 m walk time and UAG (Up And Go) improved significantly in the second assessment compared to the first assessment (*p* = 0.015 in 10 m walk and *p* < 0.001 in UAG). However, the improvement in gait performance did not correlate with the improvement in the TWSTRS assessment (Spearman’s rank correlation coefficient for 10 mW R = 0.113; *p* = 0.525 and for UAG R = 0.050; and *p* = 0.778).

### 2.3. Pain

In terms of pain, a marked improvement in the VAS (Visual Analog Scale) following the administration of botulinum toxin was observed (mean VAS score in first assessment was 5.03 [SD] = 2.29; second assessment 3.12 [SD] = 2.51). However, the reduction in the VAS score in the second compared to the first assessment did not reach the level of statistical significance (*p* = 0.077).

### 2.4. Cognitive Functions

There was a statistically significant change (*p* < 0.001) in the total MoCA score after BoNT treatment. A series of statistically significant differences were identified in the results of the MoCA test, with respect to individual cognitive domains. Firstly, it was observed that visuoconstructional skills improved in the second assessment. Furthermore, statistical analysis revealed significant improvements in the language and memory subscales (*p* = 0.013 and *p* = 0.002, respectively).

### 2.5. Depressive Symptoms

In the study group, the median BDI (Beck Depression Inventory) score, which is a measure of the severity of depressive symptoms, revealed a statistically significant decrease in the second examination in comparison with the baseline (*p* = 0.0004). The median score in the first examination was 8.5 (range: 0–27 points) and the median score in the second examination was 6.0 (range: 0–33 points). A total of 29.41% of patients included in the study were found to have BDI scores exceeding the cut-off score for at least minimal depression in the baseline assessment. In the second assessment, a significant improvement was found with only 11.76% of patients with scores indicating signs of depression (10 vs. 4 out of 34).

### 2.6. Anxiety Symptoms

With regard to anxiety, no statistically significant improvements after BoNT treatment were observed in the LSAS (Liebowitz Social Anxiety Scale) total score and avoidance subscale (*p* = 0.109 and *p* = 0.411, respectively), although a trend indicating a reduction in anxiety symptoms was identified in the LSAS anxiety subscale (*p* = 0.058) across social interaction and performance situations.

### 2.7. Sleep Disturbances

The AIS (Athens Insomnia Scale) score improved significantly after BoNT treatment (mean AIS score at baseline was 7.41; [SD] = 0.86 vs. mean AIS score in the second examination was 6.03; [SD] = 0.78; and *p* = 0.022) in the cervical dystonia patients group.

### 2.8. Dystonia Severity and Non-Motor Symptoms

There was a statistically significant effect of botulinum toxin injection on motor functioning in the TWSTRS scale. However, there was no statistically significant correlation between dystonia severity (TWSTRS severity subscale scores) and the severity of cognitive functions (MoCA), depressive symptoms (BDI), anxiety symptoms (LSAS total and both LSAS subscales), or sleep disturbances (AIS) either in the first or in the second examination (Table 5).

### 2.9. Disease Duration and Treatment Effectiveness

The disease duration did not have an impact on treatment effectiveness and did not influence improvement in the outcome in the second study in terms of cognitive function, depressive symptoms, anxiety symptoms, pain, sleep disturbances, or gait performance (Spearman’s rank order correlation *p* > 0.05).

## 3. Discussion

In the literature, there are limited number of studies that investigated both motor and non-motor symptoms of dystonia and the effectiveness of BoNT on these characteristics [8]. It was hypothesized by the authors of the present study that, in addition to its motor effect on the affected muscles, BoNT treatment also exerts a “Non-Motor Effect” defined as an improvement of NMS after BoNT treatment. It is evident that the evaluation of the NMS of dystonia without addressing the severity of the motor symptoms of the disease would be unreliable, as motor and non-motor symptoms of the disease form a complex interplay. In order to verify the research hypothesis, a comprehensive clinical and functional assessment of patients with cervical dystonia treated with BoNT has been performed to evaluate the prevalence and severity of NMS in cervical dystonia patients and to determine the effect of botulinum toxin treatment on the examined motor and non-motor symptoms.

Several mechanisms have been proposed to explain the beneficial effects of BoNT on NMS, which extend well beyond the neuromuscular junction. Evidence from preclinical and clinical studies suggests that BoNT may exert central neuromodulatory effects via retrograde axonal transport. This allows the substance to reach central structures and modulate neurotransmitter release. Another possible path is through the reduction in abnormal sensory input from dystonic muscles, which can normalize dysfunctional sensorimotor integration. Neurophysiological studies in humans have shown that BoNT can alter cortical excitability and sensorimotor integration [12], and animal models have demonstrated improvements in affective behaviors following central administration [13]. Regarding cervical dystonia, recent findings suggest that BoNT treatment may contribute to improvements in mood and cognitive function, indicating broader neuropsychiatric benefits [9,15]. Additionally, an improvement in motor symptoms may lead to better psychosocial functioning, reduced stigma, and an improved quality of life. These observations support the complex therapeutic profile of BoNT.

The TWSTRS has been identified as the most widely accepted scale for assessing the severity of dystonia. In the present research, the TWSTRS total score (*p* < 0.0001), as well as the TWSTRS severity and disability subscales (*p* < 0.0001 in both subscales), improved in the second examination compared to the pre-treatment score. The forementioned results are in line with most of the studies assessing the influence of BoNT on motor symptoms [17]. What is interesting is that the parameters assessing mobility and gait also improved significantly, but the improvement did not correlate with the improvement in TWSTRS. In other words, there was no correlation between the change in TWSTRS score between pre- and post-treatment and the reduction in walking times per 10 m (*p* = 0.525, R = 0.113) and the reduction in UAG times (*p* = 0.778, R = 0.050). This study makes a contribution to the existing body of literature on the impact of BoNT on ambulatory function.

In terms of pain, a marked improvement following the administration of the botulinum toxin was observed. However, the reduction in VAS score in the second compared to the first assessment did not reach the level of statistical significance (*p* = 0.077). This tendency is compatible with the findings of the majority of studies conducted to date, which have demonstrated significant improvements in pain levels following botulinum toxin treatment [9,18]. However, this discrepancy can be attributed to the methodology employed to assess pain intensity. For instance, in this study, the TWSTRS pain subscale improved significantly in the second assessment (*p* < 0.0001), which aligns with other studies using TWSTRS in pain assessment [9].

Whereas the motor assessment of patients with dystonia is well established, a variety of assessment scales are used to examine non-motor symptoms in research studies, and there is no single assessment tool to date. In the domain of cognitive assessment, the MoCA is a widely used screening tool for mild cognitive impairment and early dementia [19]. Sensitivity to subtle cognitive deficits makes the scale suitable for research requiring a brief but comprehensive measure of cognitive performance. In the present study, MoCA was employed to determine cognitive deficits, and it is in line with prior studies on cognition in dystonia [20,21]. In the current study, a substantial improvement in the MoCA score was observed 4–6 weeks after BoNT treatment (*p* < 0.001). This finding is consistent with other research concerning the cognitive function improvement following botulinum toxin treatment by Bailey et al. [20] and Defazio et al. [21]. This is a multifaceted phenomenon that can be explained by the fact that by mitigating painful and distracting muscle contractions, the patient is able to concentrate on their cognitive tasks. It is also important to note that the abnormal head posture and head tremor associated with cervical dystonia can have an impact on visuospatial functioning. It has been demonstrated by O’Connor et al. [22] that symptom relief after BoNT treatment can lead to improvements in these functions. It is also hypothesized that central actions could also play a role in cognitive functioning, as BoNT may influence the activity in cortical and subcortical brain areas that overlap with cognitive circuits [12]. It is not yet established whether the subtle cognitive dysfunction found in cervical dystonia patients is an independent non-motor variable and can be attributed to the disease itself or if it is a result of an overlap of motor and other non-motor symptoms like depression, anxiety, and pain [22,23].

The assessment of depressive symptoms in patients with dystonia has been largely studied, but the tools used for this assessment varied [8,9,15,24]. Several scales largely used for mood disturbances in dystonia, like the BDI or Hospital Anxiety and Depression Scale (HADS), are self-assessment tools. Other scales like the Hamilton Depression Rating Scale (HAM-D) or Montgomery Asberg Depression Rating Scale (MADRS) are clinician-rated. In the present study, the BDI was used to determine the severity of depressive symptoms. The BDI aligns with DSM-IV criteria for major depressive disorder and demonstrates strong internal consistency (Cronbach’s α ≈ 0.90) and validity across different clinical and neurological populations [25]. A statistically significant decrease in the severity of depressive symptoms was found after botulinum toxin treatment. This finding is in accordance with preceding research on the severity of depressive symptoms following botulinum toxin administration by Costanzo et al. and by Ceylan et al. [9,15].

Similarly to depression, anxiety disorders in dystonia have been of considerable interest to researchers [9,15,26]. In this area, the assessment tools varied from self-assessments like HADS and the State-Trait Anxiety Inventory used in the research by Ceylan et al., Dec-Cwiek et al., and Sugar et al., to clinician-rated scales, e.g., Hamilton Anxiety Rating Scale (HAM-A) or MADRS. In the present study, the Liebowitz Social Anxiety Scale (LSAS) has been utilized, which is a self-assessment test that has demonstrated strong psychometric properties, including high internal consistency and test–retest reliability, and is commonly used in both clinical and research settings to assess social anxiety symptoms and treatment outcomes [27]. LSAS has been previously used in dystonia patient populations assessing their social anxiety [2]. In the current study, no statistically significant improvements after BoNT treatment were observed on the social anxiety scale, although a trend indicating a reduction in anxiety symptoms was identified in one of the LSAS subscales (*p* = 0.058), indicating a subtle improvement in social interaction and performance situations. A number of studies conducted by other researchers have indicated a significantly more pronounced improvement following treatment with the botulinum toxin [9,15]. However, it should be noted that there are also studies that have demonstrated inconclusive results in this regard [8].

Previous studies have not yielded a conclusive effect of BoNT on sleep disorders, with results varying depending on the assessment tools used [9,28]. In the present study, the Athens Insomnia Scale was used and an improvement following BoNT treatment was demonstrated. In a Polish validation study, it was confirmed that the AIS is an effective tool for the assessment of insomnia. Very good psychometric properties of AIS, questions related to various domains of sleep, and brief administration make it a useful tool in clinical practice and research. It is noteworthy that the study group did not differ significantly in terms of AIS scores when compared with the control group (*p* = 0.082).

To the best of our knowledge, this study is the first to assess such a comprehensive evaluation of BoNT’s effect on motor as well as non-motor symptoms of dystonia, with a specific focus on neuropsychological and functional improvement. It is important to note that even though the effects of BoNT on NMS are considerable, the impact on various symptoms was not equal.

Despite its valuable contributions, this study is not without limitations. A primary consideration is the relatively small size of the study cohort. However, it is important to recognize that dystonia is a rare neurological disorder, which restricts the availability of large patient populations for clinical research.

A notable limitation of the study is its lack of blinding, which introduces potential bias, and the absence of a placebo control group, which limits the generalizability of the findings. However, the placebo could not have been used due to ethical concerns that advise against its utilization in cases where standard care is available or where the administration of sham procedures may pose a risk without providing any benefit.

## 4. Conclusions

The presence of non-motor symptoms alongside motor manifestations in cervical dystonia contributes to the complex clinical picture of the disease. It is essential to address both domains to effectively improve patients’ quality of life. The results of the present study prove that the administration of BoNT to the affected muscles in cervical dystonia exerts effects that extend beyond the purely motor domain, influencing NMS such as pain, mood, and cognitive function. This phenomenon has been called the “Non-Motor Effect” of botulinum toxin treatment and highlights the broader therapeutic potential of BoNT in cervical dystonia. However, these findings require confirmation in studies involving larger, multicenter cohorts. Further investigation into the impact of BoNT on non-motor symptoms may support the development of more comprehensive and individualized therapeutic goals. In clinical practice, this underlines the importance of a multidisciplinary approach that includes the regular assessment and management of non-motor symptoms as part of routine care in cervical dystonia.

## 5. Materials and Methods

### 5.1. Study Design and Bioethics

A prospective, controlled, observational, and clinical study was performed between 1 July 2023 and 30 June 2024. The Strengthening the Reporting of Observational Studies in Epidemiology (STROBE) cohort reporting guidelines were used for this study. The study had been approved by the local research ethics committee of Poznan University of Medical Sciences before the experiment was started. The protocol number attributed by the ethics committee was 525/23, and the date of approval was 29 June 2023. The research has been conducted in accordance with the principles of the Helsinki Declaration and participation in the study was voluntary. The study’s design and purpose were explained to all participants, after which written consent was obtained from each individual.

### 5.2. Study Participants

A total of 34 patients aged 18–65, diagnosed with idiopathic cervical dystonia were recruited for the study among participants of the Polish National Health Care Treatment Program of focal dystonia with botulinum toxin (standard qualification under the conditions set out by the National Health Fund Program, made by a specialist in neurology with experience in the treatment of BoNT). All of the included participants have already been treated with botulinum toxin before; there were no treatment naïve patients. The main exclusion criteria were: known allergies/hypersensitivity to the neurotoxin complex or any of the ingredients of botulinum toxin A; a history of Deep Brain Stimulation implantation; and comorbidities that make it impossible to carry out the studies planned in the research protocol (NYHA [New York Heart Association scale] heart failure above II/III, mobility disorders like paresis or walking assistance required, chronic kidney disease [GFR < 60 mL/min/1.73 m^2^], clinically significant visual disturbances confirmed by an ophthalmological examination, lack of logical verbal contact, mental illnesses that make it impossible to conduct neuropsychological tests, and people who do not consent to the examination.) The ability to make logical verbal contact in order to obtain informed consent was mandatory to participate in the study.

An age- and gender-matched control group was recruited among volunteers without dystonia. The exclusion criteria were coherent with those for the study group. The sample size was planned at the study design stage based on the literature data and calculated using G*Power version 3.1.9.2. The calculations were performed for the Wilcoxon–Mann–Whitney test (for two independent groups) with an assumed significance level of α = 0.05, statistical power of (1 − β) = 0.80, and a medium effect size (Cohen’s d = 0.5). The recommended total number of participants was 64 (32 in each group).

### 5.3. Methods—Clinical Measurement

Study participants with dystonia were examined at two time points: on the day of the planned BoNT injection—before the treatment procedure (first assessment)—and within 4 to 6 weeks after BoNT injection (second assessment). Four weeks after injection is considered the “peak effect” time point in most of the studies and clinical trials [29]. The control group was assessed at one time point.

The study group underwent a detailed neurological examination with the assessment of the dystonic pattern according to Col-Cap concept [30] and TWSTRS scale assessment performed by a neurologist with experience in movement disorders and botulinum toxin treatment. Both the study group and the control group were analyzed in terms of cognition, depression, anxiety disorders, sleep disturbances, and gait performance. Additionally, all participants were asked to complete the questionnaire to assess social status and medical history. The tests and measures that were used are described below.

#### 5.3.1. Questionnaire

The study questionnaire was completed by the participants after a careful and precise introduction by the physician. The questionnaire consisted of a basic social status survey (age; sex; level of education; and type of job), as well as questions concerning medical history. The later questions were included to determine the following: comorbidities; medications used in chronic and short-term treatment; and for the patients: time of dystonia diagnosis and self-assessment of the main motor and non-motor symptoms (e.g., tremor, “sensory trick”, depression, anxiety, sleep disturbance, pain, and patient’s perception of the effectiveness of the BoNT treatment).

The control group was assessed with the same parameters as the study group, except for questions concerning dystonia. All participants were asked about their chronic medication use. Individuals taking chronic painkillers, antidepressants, anxiolytics, or sleep medications were excluded from the study because their inclusion would have significantly affected the results.

#### 5.3.2. Cervical Dystonia Severity Assessment

TWSTRS has been employed to evaluate the severity of dystonia. An evidence-based review of dystonia rating scales identified TWSTRS as one of two scales recommended for the assessment of the severity of cervical dystonia [31]. The scale consists of three subscales. The first one assesses muscular symptoms (range and duration of neck and head movements, effect of sensory tricks and shoulder elevation). The second subscale describes the patient’s disability in terms of difficulties with daily activities. The third subscale is used for assessing pain.

#### 5.3.3. Functional—Mobility Assessment

Mobility and gait performance have been examined using a 10 m walk time (10MWT) and Timed Up And Go test (UAG). 10MWT is an important diagnostic instrument in the identification of neuromuscular and neurodegenerative conditions [32]. The test measures the time (in seconds) needed to walk a distance of 10 m, allowing for the calculation of the gait speed. The task was performed three times, and the mean of three results was used. The UAG test is a validated and reliable measure of functional mobility, commonly used to assess individuals at risk of health deterioration and to evaluate responses to interventions aimed at improving function and quality of life [33]. The test was assessed by checking the time from standing up out of a chair, walking 3 m, turning around, walking back to the chair, and sitting down and is shown in seconds. Similarly to 10MWT, the mean of three results was used for the analysis.

#### 5.3.4. Cognitive Assessment

The MoCA has been used to assess cognitive functions. The MoCA evaluates multiple cognitive domains, including attention, executive function, memory, language, visuospatial skills, and orientation in a 30-point scale administered in approximately 10 min, with a cut-off score of 26 and below indicating cognitive impairment [19]. It is evident that the repetition of cognitive tasks within a brief interval could potentially impact the outcomes due to the learning effect. In order to minimize this phenomenon in the study group, two alternative versions of the MoCA (8.1 and 8.2) questionnaire have been used to assess cognitive functions before and 4–6 weeks after BoNT treatment. While learning effects are most significant with memory and executive function assessments, they are minimal or absent in mood and symptom self-reports, such as anxiety, sleep disturbances, and pain.

#### 5.3.5. Depression Assessment

The BDI-II has been used to assess depressive symptoms. The BDI is a widely used self-administered inventory that measures the severity of depressive symptoms. Developed by Beck et al. [34], the scale consists of 21 items, each scored on a four-point Likert scale (0–3), with total scores ranging from 0 to 63. Higher scores reflect greater severity of depressive symptoms, with standard cut-off scores indicating minimal (0–13), mild (14–19), moderate (20–28), and severe (29–63) depression.

#### 5.3.6. Anxiety Measurement

The LSAS is a self-reported measure designed to assess social anxiety disorders. It consists of 24 items, evaluating both anxiety (fear) and avoidance across social interaction and performance situations. Each item is rated on 4-point scale (0–3), with higher scores indicating greater anxiety severity, with a cut-off score of 30 points or more.

#### 5.3.7. Sleep Disturbances Assessment

To assess sleep disturbances, the AIS was implemented. It is a self-assessment, eight-item scale that allows the measurement of insomnia symptoms based on the criteria of the ICD-10 and consists of eight items: the first five relate to sleep induction, awakenings during the night, final awakening, total sleep duration, and sleep quality, and the last three items refer to well-being, functional capacity, and sleepiness during the day. In the original validation studies of AIS, it was shown to have high reliability and accuracy. A total score of 8 or more is considered to be, with high probability, a predictor of inorganic insomnia according to ICD-10 criteria [35].

#### 5.3.8. Pain Assessment

The VAS has been used, as it is a validated subjective measure of acute and chronic pain. Scores have been recorded by making a handwritten mark on a 10 cm line which represents a continuum between “no pain” and “worst pain” [36].

### 5.4. Statistical Analysis

Statistical analysis was performed using Statistica 13 software (StatSoft, Kraków, Poland). Descriptive statistics were calculated and presented according to the type of variable: means with standard deviations for continuous variables, medians with ranges for ordinal variables, and counts with percentages for nominal variables. The normality of data distribution was assessed using the Shapiro–Wilk test. Differences between the control and study groups were evaluated using Student’s *t*-test for independent samples when the data followed a normal distribution. In cases of non-normal distribution and for ordinal variables, the Mann–Whitney U test was used, while the Pearson’s chi-square test was applied for nominal variables. To compare changes between the first and second measurements in the study group, the paired Student’s *t*-test was used for normally distributed data, and the Wilcoxon signed-rank test was applied when the data were not normally distributed or were ordinal. Correlation analyses were performed using the Spearman’s rank correlation coefficient. A *p*-value of <0.05 was considered statistically significant.

## Figures and Tables

**Table 1:** Demographic characteristics of the studied population (study and control group).

Characteristics	Study Group (n = 34)	Control Group (n = 33)
Age in years (mean ± SD)	54	52
Females (n; %)	28; 82	29; 88
Education (n)		
- <8 yrs	- 2	- 0
- 8–12 yrs	- 15	- 14
- >12 yrs	- 17	- 19
Right-handed (%)	97	88
Disease duration (yrs ± [SD])	1	−
Presence of tremor (n; %)	25 (64)	−
Presence of “sensory trick” (n; %)	14 (41)	−

**Table 2:** Treatment characteristics of patients with cervical dystonia.

	n (%)	Mean Dose per Patient (IU)
Type of botulinum toxin		
- Onabotulinum toxin	16 (47)	695
- Abobotulinum toxin	15 (44)	228
- Incobotulinum toxin	3 (9)	233
The presence of side effects after BoNT	3 (9)	do not apply
Number of muscles injected with BoNT		
- 2 muscles	2 (6)	do not apply
- 3 muscles	2 (6)	do not apply
- 4 muscles	16 (47)	do not apply
- ≥5 muscles	14 (41)	do not apply

**Table 3:** Comparison of the study group at baseline and control group in terms of cognition (MoCA), depression (BDI), anxiety (LSAS total score and LSAS anxiety and avoiding subscales), sleep disturbances (AIS), and gait performance (10 m walk time [s] and UAG time [s]). *p* values in bold are statistically significant.

	Study Group at Baseline n = 34			Control Group n = 33			
Parameter	Mean (SD)	Median (Range)	Confidence Interval	Mean (SD)	Median (Range)	Confidence Interval	*p*-Value
MoCA	26.18 (2.48)	26.00 (20.00–30.00)	25.31–27.04	28.88 (1.17)	29.00 (26.00–32.00)	28.47–29.29	**<0.001**
BDI	8.26 (6.60)	7.50 (0–27.00)	5.96–10.57	5.48 (4.29)	4.00 (0–19.00)	3.96–7.01	**0.026**
LSAS total score	44.85 (28.84)	32.50 (6.00–116.00)	34.79–54.91	21.42 (16.19)	18.00 (0.52)	15.68–27.16	**<0.001**
LSAS anxiety	24.26 (15.64)	16.50 (6.00–57.00)	18.81–29.72	10.94 (8.89)	8.00 (0–28.00)	7.79–14.09	**<0.001**
LSAS avoiding	20.71 (14.10)	16.50 (0–59.00)	15.79–25.63	10.48 (8.44)	11.00 (0–26.00)	7.49–13.48	**0.003**
AIS	7.41 (5.04)	6.00 (1.00–20.00)	5.65–9.17	5.36 (3.91)	4.00 (0–15.00)	3.98–6.75	0.082
10 m walk	5.79 (0.96)	5.91 (4.05–7.18)	5.46–6.13	4.76 (0.43)	4.75 (3.98–5.81)	4.61–4.92	**<0.001**
UAG	6.02 (0.98)	5.93 (3.80–7.62)	5.68–6.36	4.67 (0.42)	4.68 (4.02–5.40)	4.52–4.82	**<0.001**

**Table 4:** Comparison of the results in baseline and second assessment (pre- and post-BoNT treatment) in terms of motor and non-motor symptoms of cervical dystonia. The severity of dystonia (TWSTRS), pain due to disease (VAS), gait performance (10 m walk time and UAG time), cognition (MoCA), depression (BDI), anxiety (LSAS total score and LSAS anxiety and avoiding subscales), and sleep disturbances. Results of 10 mW and UAG are presented as the mean with standard deviation in brackets. All other results are presented as a median with range (minimum–maximum) in brackets. *p* values in bold are statistically significant. Total n = 34.

Parameter	Baseline Assessment n = 34	Second Assessment n = 34	*p*
TWSTRS	38 (9–64)	22 (5–53)	**<0.0001**
- severity	16 (5–24)	9 (1–22)	**<0.0001**
- disability	13.5 (4–25)	7 (4–22)	**<0.0001**
- pain	9.5 (0–18)	5 (1–15)	**<0.0001**
VAS	5 (0–9)	3 (0–8)	0.077
10 mW	5.79 (0.96)	5.36 (0.14)	**<0.001**
UAG	6.02 (0.98)	5.81 (0.97)	**0.015**
MoCA	26 (20–30)	28 (23–30)	**<0.001**
BDI	8.5 (0–27)	6 (0–33)	**0.0004**
LSAS	32.5 (0–116)	31.5 (1–111)	0.109
AIS	6 (1–21)	5 (0–21)	0.022

**Table 5:** Spearman’s rank correlation between TWSTRS severity subscale and the results of NMS scales in the baseline and in the second assessment (n = 34).

	Baseline Assessment		Second Assessment	
Correlation	R	*p*	R	*p*
TWSTRS severity and MoCA	−0.295	0.090	0.012	0.948
TWSTRS severity and BDI	−0.158	0.372	0.210	0.233
TWSTRS severity and LSAS	0.144	0.418	0.249	0.155
TWSTRS severity and LSAS anxiety	0.127	0.473	0.098	0.581
TWSTRS severity and LSAS avoiding	0.144	0.416	0.262	0.134
TWSTRS severity and AIS	−0.138	0.435	0.243	0.167

## Data Availability

The original contributions presented in this study are included in the article/Supplementary Material. Further inquiries can be directed to the corresponding author(s).

## Supplementary Materials

The following supporting information can be downloaded at: https://www.mdpi.com/article/10.3390/toxins17080396/s1, File S1: “Patient’s Questionnaire”.

## Abbreviations

The following abbreviations are used in this manuscript:

BoNT	Botulinum Toxin Type A
CD	Cervical Dystonia
NMS	Non-Motor Symptoms
TWSTRS	Toronto Western Spasmodic Torticollis Rating Scale
UAG	Up And Go
VAS	Visual Analog Scale
MoCA	Montreal Cognitive Assessment
BDI	Beck Depression Inventory
LSAS	Liebowitz Social Anxiety Scale
AIS	Athens Insomnia Scale
HAM-D	Hamilton Depression Rating Scale
MADRS	Montgomery Asberg Depression Rating Scale
HAM-A	Hamilton Anxiety Rating Scale
NYHA	New York Heart Association scale
10MWT	10-meter walk time
