# Supplementary material for: Is There a “Non-Motor Effect” of Botulinum Toxin Treatment in Cervical Dystonia in Addition to Its Effects on Motor Symptoms?
Source: Toxins (Basel). 2025 Aug 6;17(8):396. doi: 10.3390/toxins17080396 (PMC12390245; doi:10.3390/toxins17080396)
Supplement: Supplementary file 1 [file toxins-17-00396-s001.zip › toxins-3753236-supplementary.pdf]

**"COMPREHENSIVE ASSESSMENT OF PATIENTS WITH DYSTONIA TREATED WITH BOTULINUM TOXIN" – Patient's Questionnaire for Research Purposes**

We kindly ask you to complete this questionnaire. All collected data will be anonymized and used solely for scientific research purposes. We assure you that your data will be stored securely, in full compliance with personal data protection regulations, and will not be shared with any individuals or institutions not directly involved in the research project.

1. Full name: ___________________________________

2. Gender:
☐ Female ☐ Male

3. Age:
☐ Under 18 ☐ 18–30 ☐ 31–40 ☐ 41–50 ☐ 51–60 ☐ Over 60

4. Highest level of education:
☐ No education or incomplete primary
☐ Primary education
☐ Vocational education
☐ Secondary education
☐ Higher education – Bachelor's degree
☐ Higher education – Master's degree
☐ Higher education – Doctoral or higher

5. Total years of education:
☐ Less than 8 years ☐ 8–12 years ☐ More than 12 years

6. What type of work do you perform?
☐ Manual ☐ Mental ☐ Other

7. Which category best describes your employment status:
☐ Employed, working 1–39 hours/week
☐ Employed, working 40+ hours/week
☐ Unemployed, seeking work
☐ Unemployed, not seeking work
☐ Retired
☐ Disabled, unable to work

8. Place of residence:
☐ Rural ☐ Town < 50,000 ☐ City > 50,000

9. Year of dystonia diagnosis: __________

10. Handedness:
☐ Right-handed ☐ Left-handed

11. Do you undergo regular rehabilitation?

☐ Yes:

a) Manual therapy / massage / osteopathy

b) Physical therapy (laser, TENS, magnetotherapy, cryotherapy, ultrasound, etc.)

☐ No

12. Have you ever received psychiatric treatment?

☐ No ☐ Yes – Reason: ______________________

13. Are you currently receiving psychiatric treatment?

☐ No ☐ Yes – Reason: ______________________

14. Chronic illnesses (check all that apply):

☐ Parkinsonism ☐ Hypertension ☐ Heart disease

☐ Venous insufficiency ☐ Diabetes ☐ Thyroid disorders ☐ Asthma/COPD

☐ Cataract ☐ Kidney disease

☐ Gout ☐ Liver disease ☐ Epilepsy ☐ Stroke

☐ Polyneuropathy ☐ Joint/spinal disorders ☐ Other: ____________

15. Current medications: ____________________________________

16. Are you suffering from the following symptoms (check all that apply):

☐ Tremor ☐ Pain ☐ Sleep issues ☐ Depression ☐ Anxiety

17. Effectiveness of botulinum toxin treatment:

A. Head twisting/tremor:

☐ Very effective ☐ Effective ☐ Minimally effective ☐ Ineffective ☐ Do not apply

B. Pain relief:

☐ Very effective ☐ Effective ☐ Minimally effective ☐ Ineffective ☐ Do not apply

C. Sleep improvement:

☐ Very effective ☐ Effective ☐ Minimally effective ☐ Ineffective ☐ Do not apply

D. Mood improvement:

☐ Very effective ☐ Effective ☐ Minimally effective ☐ Ineffective ☐ Do not apply

E. Anxiety reduction:

☐ Very effective ☐ Effective ☐ Minimally effective ☐ Ineffective ☐ Do not apply

18. Visual Analogue Scale (VAS). Please indicate your current level of pain by marking on the line below:

|---0---|---1---|---2---|---3---|---4---|---5---|---6---|---7---|---8---|---9---|---10---|

No pain Moderate Worst possible pain
